# Supplementary material for: Spatial attention in the moving brain: Dissociable roles of neural alpha oscillations and head rotation
Source: iScience. 2026 Aug 19;29(9):117041. doi: 10.1016/j.isci.2026.117041 (PMC13521066; doi:10.1016/j.isci.2026.117041)
Supplement: Supplementary file 1 [file mmc1.pdf]

iScience, Volume *29*

Supplemental information

Spatial attention in the moving brain: Dissociable roles of neural alpha oscillations and head rotation

Malte Wöstmann and Jonas Obleser

Supplementary information

for

Spatial Attention in the Moving Brain: Dissociable Roles of Neural Alpha Oscillations and Head Rotation

Malte Wöstmann & Jonas Obleser

Department of Psychology, University of Lübeck, Lübeck, Germany

Center of Brain, Behavior and Metabolism, University of Lübeck, Lübeck, Germany

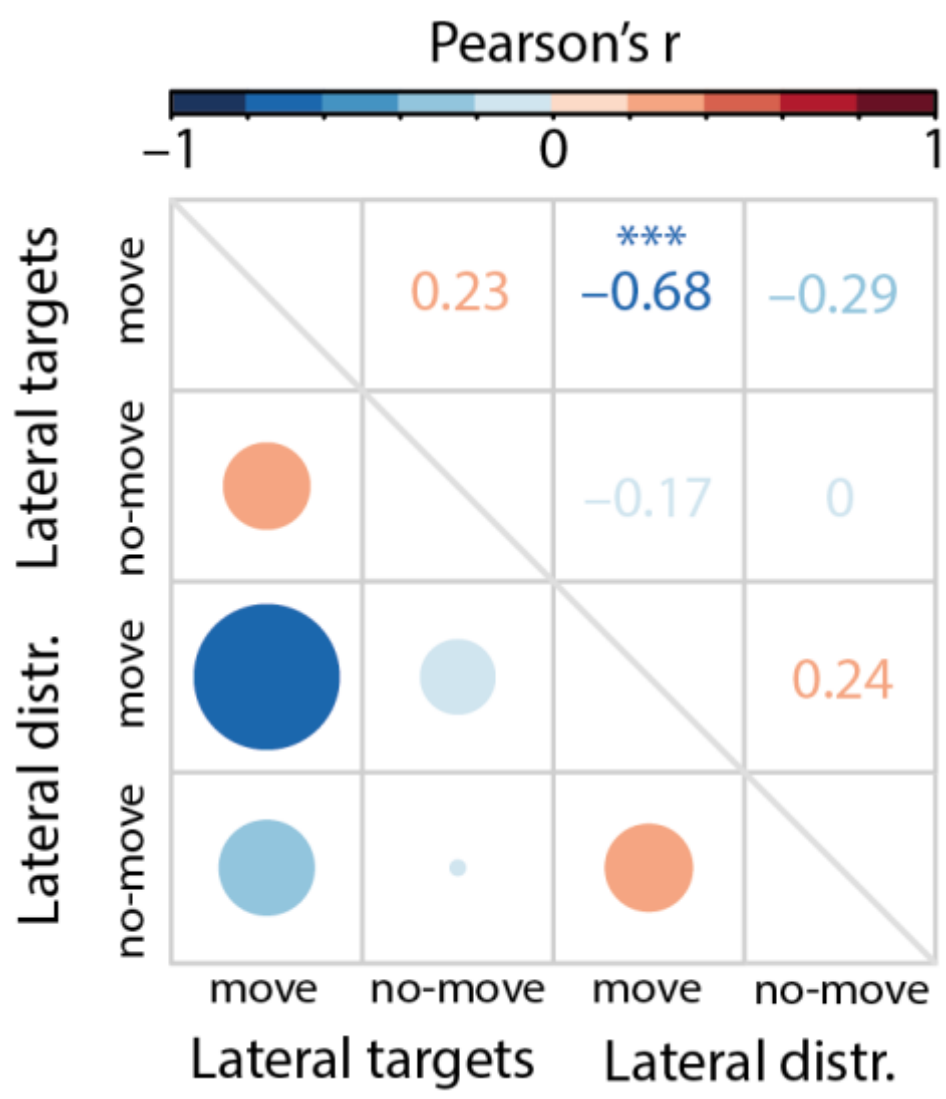

Figure S1. Heatmap shows Pearson' r for pairwise correlations of single-subject head rotation (i.e., Towardness shown in Fig. 2D in the main manuscript). Note that only the correlation between the two movement conditions was significant (lateral targets, move & lateral distractors, move; $r = –0.6794$; $p < 0.001$), indicating that participants who moved more significantly in the direction of lateral targets also moved more significantly away from lateral distractors.

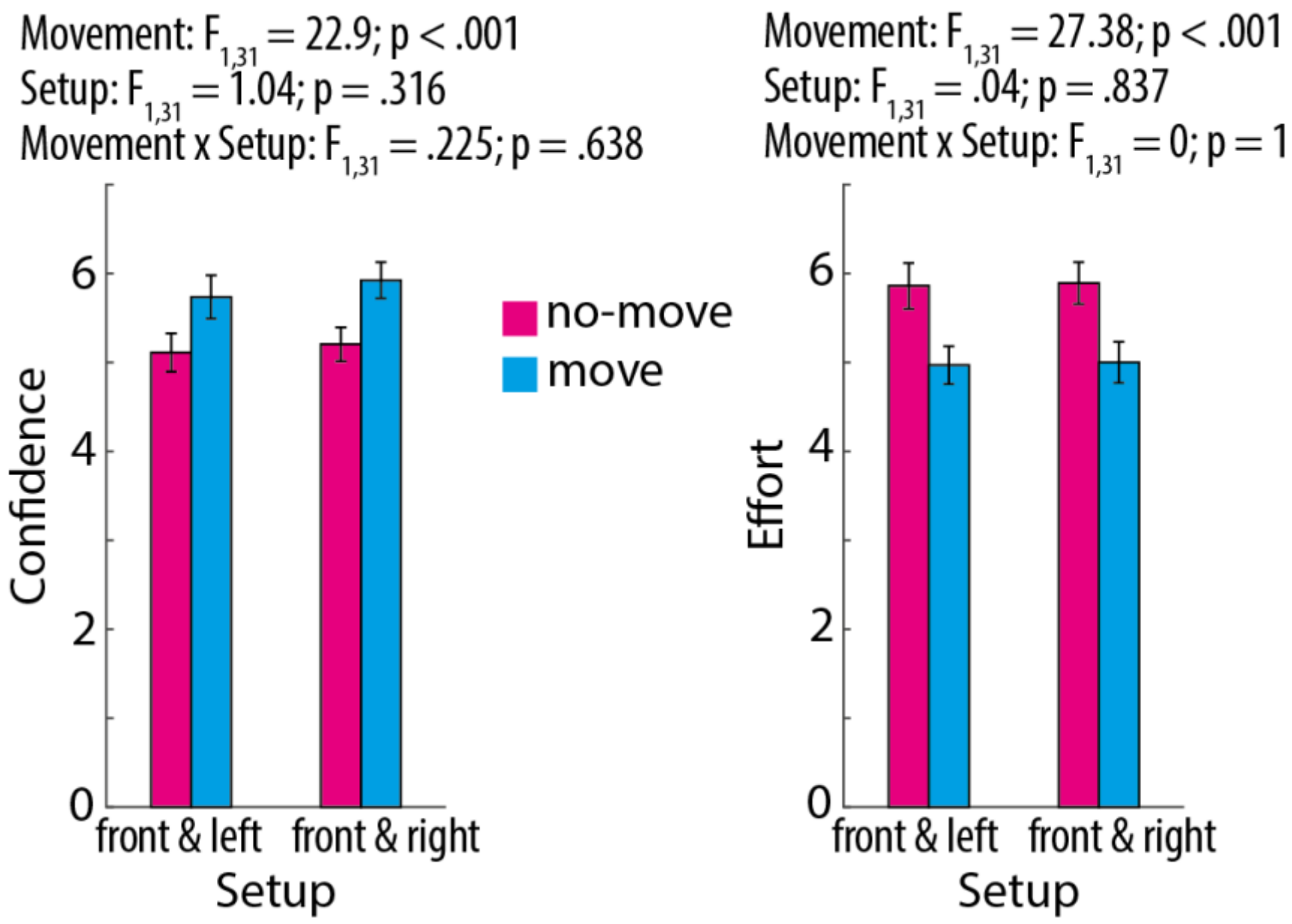

Figure S2. Confidence and effort ratings were collected in the end of each block on a scale from 1 (very low confidence / very low effort) to 10 (very high confidence / very high effort). Confidence was probed with the item "How confident are you that you reached a good performance level in this block?" Effort was probed with the item "How effortful did you experience the listening task in this block?" Results of two-way repeated measures ANOVAs with the factors movement (move vs. no-move) and loudspeaker setup (front & left vs. front & right) are shown above bar graphs. Bars show mean confidence/effort, error bars show ±1 SEM. Irrespective of the speaker setup, confidence ratings were significantly higher and effort ratings lower for blocks with movement permitted.

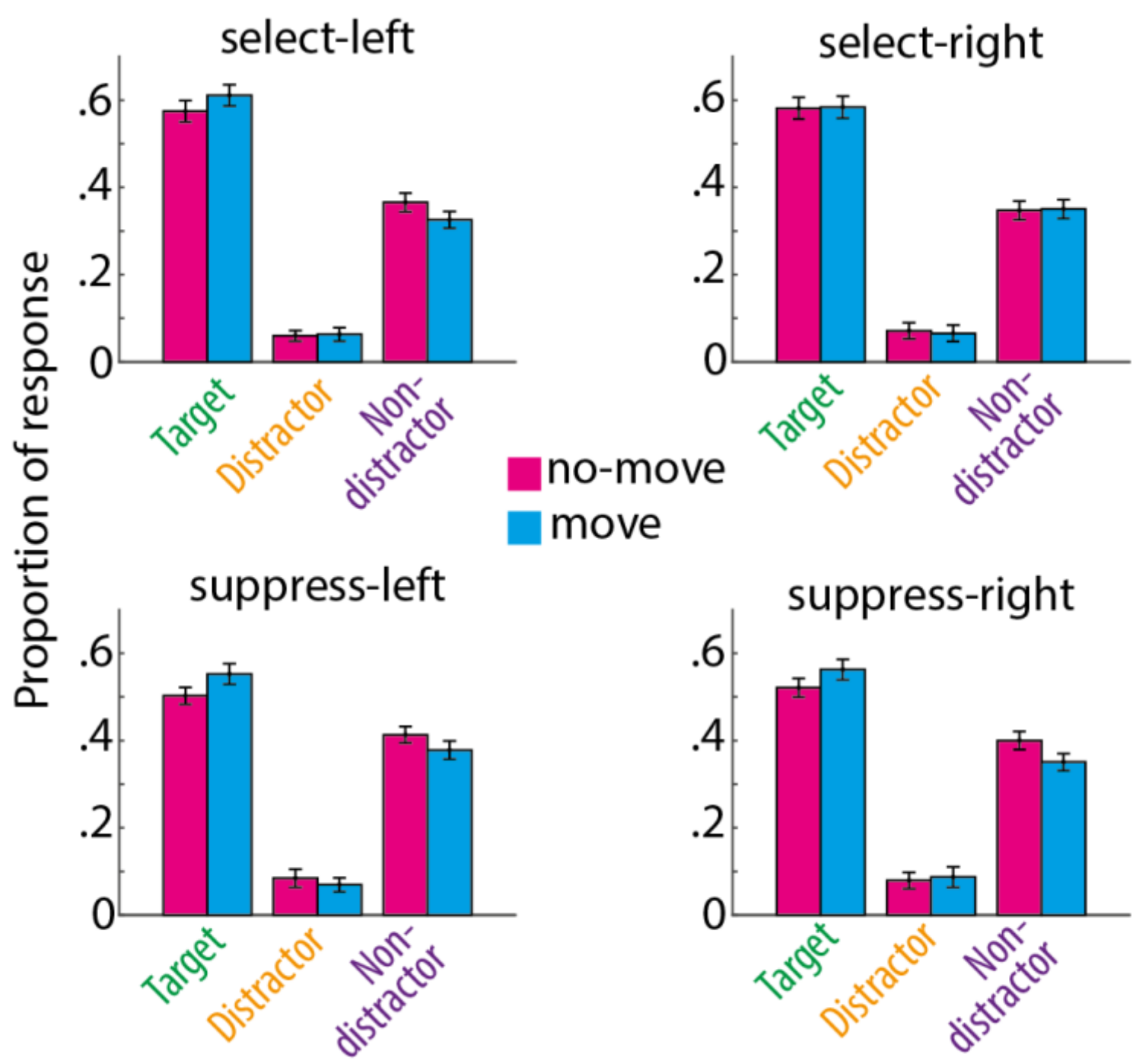

Figure S3. Bars show average proportion of responses for different response types for the four different experimental conditions. Error bars show ±1 SEM.

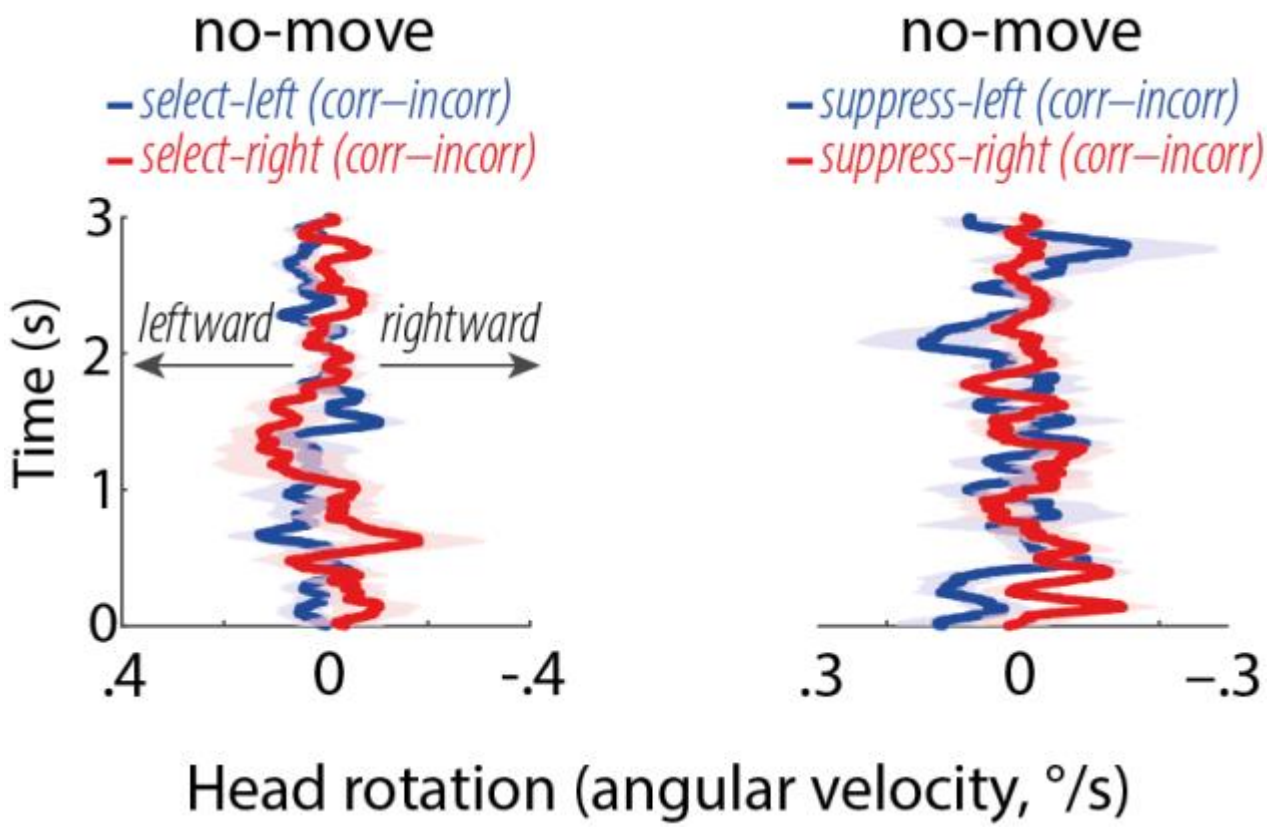

Figure S4. (**Left**) Lines and shaded areas show average angular velocity ±1 SEM for the difference of correct versus incorrect trials for trials with lateral targets and without movement permitted. (**Right**) Same as left but for trials with lateral distractors. No significant differences between correct and incorrect trials were found for these conditions (all $p > .05$, cluster-based permutation tests).
